# Pipeline Flex Embolization Device (PED Flex) for the treatment of intracranial aneurysms: Periprocedural outcomes and first-year angiographic results

**DOI:** 10.3906/sag-1906-116

**Published:** 2019-12-16

**Authors:** Şükrü OĞUZ, Ömer Naci TABAKCI, Ender UYSAL, Eser BULUT, Hasan DİNÇ

**Affiliations:** 1 Department of Radiology, Faculty of Medicine, Karadeniz Technical University, Trabzon Turkey; 2 Department of Radiology, Şişli Hamidiye Etfal Training and Research Hospital, İstanbul Turkey; 3 Department of Radiology, Kanuni Training and Research Hospital, Trabzon Turkey

**Keywords:** PED Flex, intracranial aneurysm, endovascular treatment

## Abstract

**Background/aim:**

The Pipeline Flex Embolization Device (PED Flex) is a new updated version of the PED classic that incorporates a new delivery system to allow facilitation of stent deployment, resheathing, and removal of the capture coil. This study evaluated the PED Flex in terms of the technical aspects of the procedure and first-year follow-up results.

**Materials and methods:**

This retrospective study involved prospectively collected data from May 2015 to August 2017. The primary endpoint was technical aspects of the procedure, and the secondary endpoint was first-year follow-up results.

**Results:**

Forty-nine patients with 59 target intracranial aneurysms were enrolled. Patients’ mean age was 52 years (range 21−71 years), and 31 (63.0%) were female. All aneurysms except for three were unruptured. The mean aneurysm diameter was 8 mm. Forty-seven patients with 56 aneurysms were successfully treated. Due to advancement, repositioning, and migration problems, 8 (13.1%) stents were not deployed and discharged. The total aneurysm occlusion rate was 77.0%. The mortality rate was 4.3%.

**Conclusion:**

Our experience shows that the applicability and safety of the renewed delivery system provided by PED Flex for improving device apposition and opening has been proven with one-year angiographic and clinical follow-up results.

## 1. Introduction

The Pipeline Embolization Device (PED) (Medtronic Neurovascular, Irvine, CA, USA) is one of the most widely used flow diverter stents for the treatment of intracranial aneurysms. Its safety and efficacy have been reported by the “Pipeline for Uncoilable or failed aneurysms: results from a multicenter clinical trial” (PUFS trial) [1] and the “International Retrospective Study of the PED: a multicenter aneurysm treatment study” (IntrePED study) [2], with a high rate of occlusion of aneurysms in the internal carotid artery (ICA) and a low rate of major events. The device has been routinely employed in the treatment of all intracranial aneurysms, with increasing use in small and more distal intracranial aneurysms [3,4]. The PED received the European CE mark of approval in 2008 and US Food and Drug Administration (FDA) approval in 2011 and is now considered first-generation. 

The first-generation PED can be technically challenging, particularly in an anatomically tortuous parent vessel. Problems often encountered during PED placement include difficulty in freeing the distal end of the device from a constrained capture coil, limited pushability of the delivery wire, inconsistent deployment, misplacement, stent narrowing, and inability to resheath the PED after partial deployment [5].

The Pipeline Flex Embolization Device (PED Flex) received the European CE mark of approval in March 2014 and FDA approval in February 2015. The Pipeline Flex contains a completely redesigned delivery system, while the stent device remains unchanged. The new delivery system provides the following advantages: the proximal portion has a resheathing pad to allow for recapture and repositioning of the device after partial deployment, and the distal portion has two constraining protective sleeves that allow for increased convenience of the device opening and facilitation of stent resheathing by 180° rotation upon device recapture. The pusher is also larger, with a more robust laser-cut hypotube to enhance the pushability of the device during delivery [6]. All these changes in the PED delivery system should result in easier device deployment, potentially improving procedural outcomes.

The new delivery system of the PED Flex entails numerous innovations for stent deployment. This study retrospectively evaluated 49 patients with 59 aneurysms treated using the PED Flex. The technical aspects of the procedure, such as the number of resheathing attempts, technical success outcomes, and procedure-related adverse events, were assessed as the primary endpoint, while first-year follow-up results served as the secondary endpoint.

## 2. Methods

This retrospective study from two centers was performed from May 2015 to August 2017. Forty-nine patients with 59 target intracranial aneurysms were enrolled. Written and signed informed consent was obtained from each patient. The study was approved by the institutional review board responsible for all patient data and images available in the hospital information system. All procedures were performed by interventional radiologists using a biplane flat panel angiographic system (Artis Q and Zee, Siemens, Erlangen, Germany).

In preparation for the procedure patients were treated with a dual antiplatelet regimen (clopidogrel 75 mg/prasugrel 10 mg and aspirin 300 mg daily). If an adequate platelet response was not achieved with clopidogrel, increased daily doses (e.g. 150 mg clopidogrel daily) were administered. If clopidogrel resistance was detected, clopidogrel was replaced with prasugrel. All procedures were performed under general anesthesia and systemic anticoagulation was provided during the procedure. Aneurysms were categorized according to location and size (small <10 mm, 25 mm ≥ large ≥ 10 mm, and giant >25 mm) (Table 1). We treated three ruptured aneurysms in our case series, one of which resulted in mortality due to a second bleed on the 27th day following the procedure. 

**Table 1:** Patient demographics and characteristics of target aneurysms.

Patients	n: 49
Mean age (range), years	52 (21–71)
Female	31 (63%)
Aneurysms number	n: 59
Location	
Anterior circulation	52 (88%)
Internal carotid artery	43
Petrous segment	1
Cavernous segment	6
Paraophthalmic segment	17
Supraclinoid segment	19
Anterior cerebral artery	6
Middle cerebral artery	3
Posterior circulation	7 (12%)
Size (mean diameter, mm)	8
Small, n (%)	40 (67.9%)
Large, n (%)	18 (30.5%)
Giant, n (%)	1 (1.6%)

The PED Flex was deployed through a microcatheter (Marksman; Stryker, Neurovascular, California, USA or Rebar 27; ev3/Covidien, Massachusetts, USA) using a triaxial system. Balloon angioplasty was performed in the case of incomplete stent apposition or insufficient opening of the distal or proximal portion of the stent. A suitable size of *HyperForm/HyperGlide* balloon (ev3/Covidien, Irvine, California, USA) was employed for the parent vessel. Angiographic follow-up was scheduled at 6 months, 1 year, 2 years, and 5 years after treatment and results were given according to first year follow-up.

Patient demographics and aneurysm characteristics were recorded. The primary endpoints were the technical aspects of the procedure, such as the number of resheathing attempts, use of the balloon to exact apposition, device deployment success rates, and procedure-related adverse events. The secondary endpoint was the first-year follow-up aneurysm occlusion rate based on the O’Kelly–Marotta (OKM) grading scale (A, complete filling; B, subtotal filling; C, entry remnant; and D, no filling) [7], in-stent stenosis, and morbidity/mortality rates.

## 3. Results

**Figure 1:**
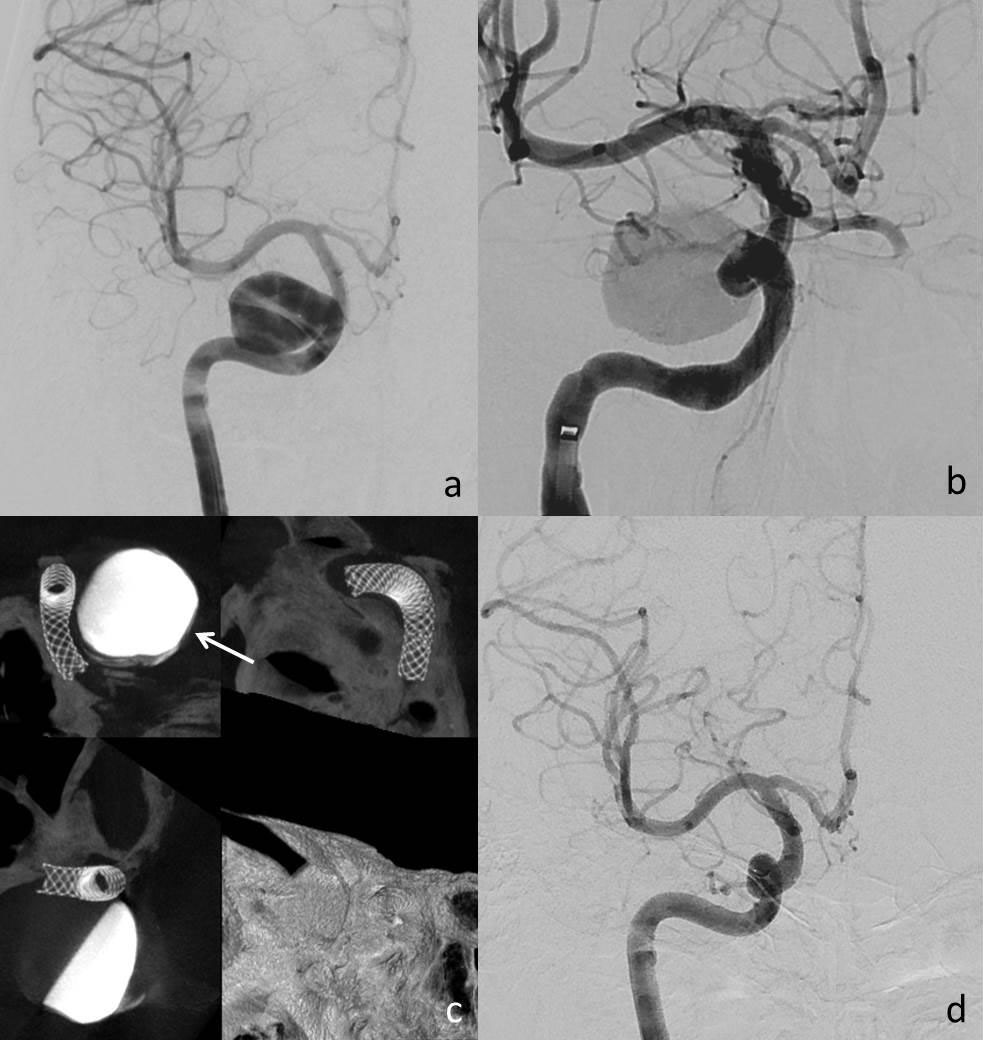
A 21-year-old man previously treated using a detachable balloon (GOLD BAL1, Balt Extrusion, Montmorency, France). a) Follow-up revealed a dissecting aneurysm 14 × 15 mm in size in the right ICA cavernous segment. b) A 4.5 × 16 mm PED Flex was deployed into the ICA, and flow into the aneurysm was markedly slower after the procedure. c) Dyna CT images show a well-opened stent and contrast medium stagnation (arrow) in the aneurysmal sac. d) A first-year control DSA image demonstrating complete occlusion of the aneurysm and full patency of the stent.

Forty-nine patients with 59 target intracranial aneurysms were included in this study. Patients’ mean age was 52 years (range 21−71 years), and 31 (63.0%) were female. All aneurysms except for three were unruptured, and ruptured aneurysms were treated two weeks subsequently for subarachnoid hemorrhage (SAH). The mean maximal aneurysm diameter was 8 mm (range 2–25 mm). Fifty-six aneurysms were saccular, two were dissecting, and one was a pseudoaneurysm, which was secondary to a previously treated carotid cavernous fistula (Figure 1). The majority of the aneurysms (n = 52) were located in the ICA. Nine were located beyond the internal carotid artery termination, and seven were located in the posterior circulation (Table 1). Forty-two patients had one aneurysm, while the other seven had multiple aneurysms. There were 23 cortical branches covered by the device, but we had 18 cortical branches in first-year follow-up (Table 2).

**Table 2:** Aneurysm occlusion rates, in-stent stenosis, and states of related cortical branches at first-year follow-up.

Aneurysm occlusion	n: 39
A (complete filling)	4 (10.2%)
D (subtotal filling)	0 (0.0%)
C (entry remnant)	5 (12.8%)
D (no filling)	30 (77.0%)
In-stent stenosis	n: 35
Fully patent	28 (80.1%)
Mild (≤ 50%)	5 (14.8%)
Moderate (50%–70%)	2 (5.1%)
Severe (≥ 70)	0 (0.0%)
Cortical branches	n: 18
Patent	11 (61.1%)
Very narrow	2 (11.1%)
Occluded	5 (27.8%)

Eight stents in eight patients could not be deployed because of a stent advancement problem or an unsuccessful resheathing attempt. Five stents encountered a problem with stent advancement into the Marksman microcatheter and were entirely removed using the microcatheter. A twist in the stent occurred in one of these cases. Resheathing or recapturing was attempted with seven devices and was successful in four. Resheathing was performed for repositioning in two stents and for insufficient opening of the distal part of the stent in two cases. Resheathing failed at two attempts at repositioning, and the stents were removed. In these cases, recatheterization was required due to retrograde proximal displacement of the stent and microcatheter, and a second stent had to be used. The stent could not be deployed in two patients, and treatment using the PED Flex was unsuccessful. The unsuccessful treatment rate was 4.1% (2/49). In conclusion, 47 patients with 56 aneurysms were treated. Altogether, 61 devices were used in this study, 53 of which were deployed to the target site. Eight (13.1%) stents could not be deployed and discharged due to advancement, repositioning, and migration problems.

Second instruments were used in 14 cases in our series in order to improve stent apposition with the balloon or to prevent early rupture with the coil. Adjunctive moderate coil packing was performed in nine cases. Balloon angioplasty was performed in five cases due to lack of stent apposition or insufficient opening of the distal/proximal part of the stent. In one of them, balloon angioplasty was performed to improve the flow of the covered cortical branches. We used the *HyperForm *or* HyperGlide *balloon (ev3/Covidien, Irvine, California, USA) in a size appropriate to the parent vessel.

Intraprocedural or periprocedural thromboembolic events occurred in four (8.1%) patients. All these presented with minor neurological deficits, and there were no clinical deficits at discharge. We had two groin complications (femoral pseudoaneurysm and retroperitoneal hematoma). One patient died due to a massive retroperitoneal hematoma secondary to postprocedural femoral access bleeding in the intensive care unit. 

**Figure 2:**
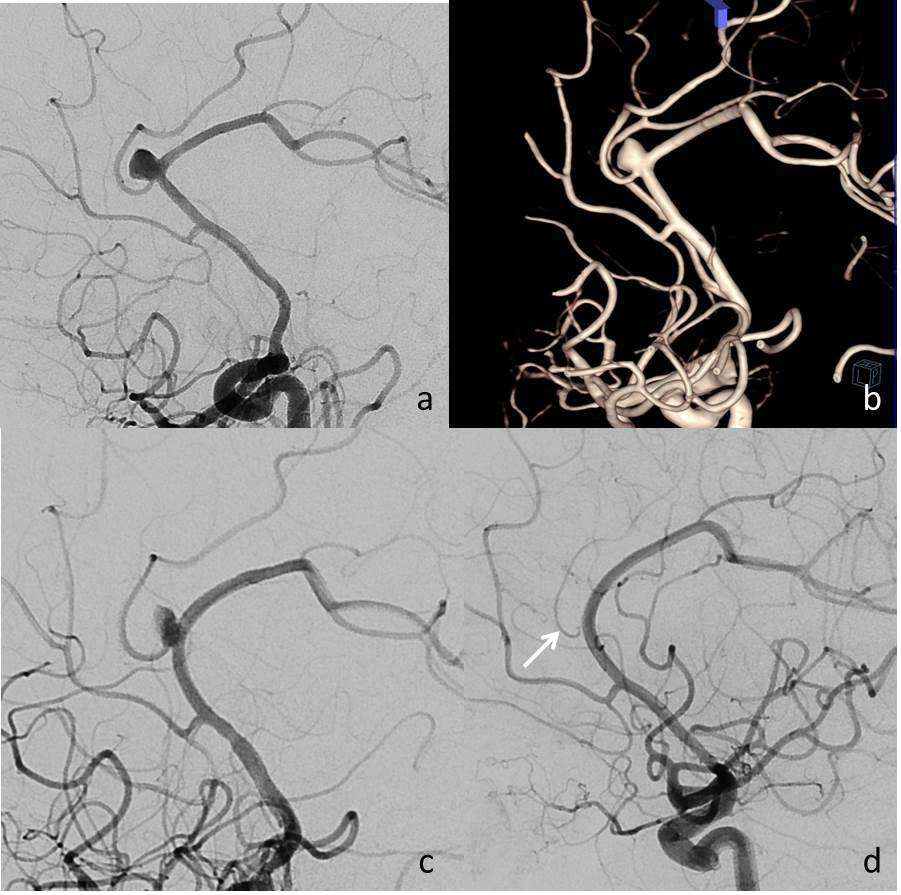
A 56-year-old woman with headache. a,b) Digital subtraction angiography (DSA) and 3D-DSA images revealed saccular aneurysm originating from the pericallosal and callosomarginal artery bifurcation. c) Angiographic image obtained immediately after deployment of the 2.5 × 14 mm PED Flex into the anterior cerebral artery A2 segment to cover the aneurysm neck. The image shows a decrease in the passage of contrast medium into the aneurysm sac following the stent placement, comparison to a. d) A first-year control DSA image demonstrating complete occlusion of the aneurysm and full patency of the stent, although diameter of the callosomarginal artery (arrow) decreased. The patient is currently asymptomatic at clinical follow-up.

The first-year follow-ups of 32 patients with 36 aneurysms were assessed using control digital subtraction angiography (DSA) images. The occlusion rate of the aneurysm, the patency of the stent, and the adjacent cortical branches of the aneurysms were evaluated. The total aneurysm occlusion rate was 77.0% at first-year follow-up. Four aneurysms were grade A (complete filling), five were grade C (entry remnant), and 30 aneurysms were grade D (no filling). Seven cases of in-stent stenosis were observed, five (14.8%) mild and two (5.1%) moderate. Eighteen cortical branches covered by the stent were evaluated. Eleven of these were patent, two were decreased in caliber (Figure 2), and five were totally occluded (Table 2).

The morbidity rate in our case series was 0.0%, although the mortality rate was 4.3%. Mortality was secondary to complications of femoral artery access and second bleed in the SAH patient, and was not related to the PED Flex or neurological ischemic complications.

## 4. Discussion

The renewed delivery system of the PED Flex has been previously reviewed in the literature [5,8−12,15,20]. While some studies evaluated the delivery system, the detail of the redesign of the delivery system was not discussed. We present our experience using the PED Flex in 49 cases with 59 aneurysms in both the anterior and posterior circulations. The successful treatment rate with the PED Flex was 95.9% (47/49). Due to anatomic vessel tortuosity, we were unable to treat two patients using the PED Flex. Fifty-seven aneurysms were treated in 47 cases with successful implantation of the PED Flex. The mean number of devices used per patient was 1.25 (59/47), and the mean number per aneurysm was 1.05 (59/56). In one large case series, the equivalent rate for the previous PED Flex per aneurysm was 1.3−1.4 [13,14]. However, the success rate of the device deployment was 86.8%. Eight (13.2%) devices were discharged or removed in our series. Five were discharged due to inability to advance through the microcatheter. We attempted to recapture or resheath seven devices and were successful in four (57%) of these. Martínez-Galdámez et al. [15] reported that resheathing was attempted with 13 devices and was successful in 12 (92.3%). Lin et al. [16] reported a 13% discharge or removal rate for the previous PED Flex. In one recent paper, the PED Flex Shield (with the same delivery system as the PED Flex) utilized a mean 1.12 devices per aneurysm, with a failure to deploy rate of 5.4% [12].

The PED Flex was introduced as a redefined delivery system consisting of new features. The new delivery system primarily provides resheathing and repositioning of the device after partial deployment. Resheathability and removal of the capture coil were considered fundamental innovations in the delivery system. These features make device deployment and apposition easier, potentially improving procedural outcomes. However, we also think that the new delivery system requires a learning curve. Pereira et al. described various technical nuances related to the use of this system. The PED Flex is delivered through simple application of an 80% pull to the microcatheter and a 20% push to the wire. Pereira et al. reported that the following two strategies can be used to initiate deployment: 1) the initial 10 mm is deployed distal to the target lesion and the partially deployed device is subsequently withdrawn to the planned landing zone; and 2) the device is navigated to the landing zone and the microcatheter is unsheathed progressively until deployment. If the distal part of the stent cannot be opened secondary to the attached protective leaves, the stent should be recaptured to invert or release the leaves and then reopened [9]. Despite these innovations developed to improve stent placement, balloon angioplasty was still required in four cases (7.0%) in our series. Martínez-Galdámez et al. [15] reported a figure of 18.0% (9/50) for adjunctive balloon use. One study comparing PED and PED Flex reported a 10.5% (6/57) angioplasty rate in the PED group compared to 2.6% (1/38) in the PED Flex group [10].

The total aneurysm occlusion rate at first-year follow-up in our series was 77.0%, the moderate/severe in-stent stenosis rate was 5.1%, and the aneurysm-related branch occlusion rate was 27.8%. The morbidity rate was 0.0% and the mortality rate was 4.3%, although mortality was not related to PED Flex (Table 2). The total aneurysm occlusion rate was corroborated by previous studies and metaanalyses [1,13,17,18]. In the PUFS study, the moderate/severe (≥50%) in-stent stenosis rate during the first year following the procedure was 2.2% (2/91). In the same study, angiographic complete occlusion rate, residual neck, and residual aneurysms were found to be 76.0%, 7.5%, and 5.7%, respectively, at 6 months follow-up, whereas the same rates were found to be 93.4%, 2.6%, and 2.6%, respectively, at 3 years follow-up [1]. Oishi et al. [19] found that complete occlusion rate, residual neck, and residual aneurysms were found to be 69.2%, 19.2%, and 9.6%, respectively, at one year follow-up in 52 of 100 large and giant unruptured aneurysms treated with PED. In the aneurysm study of pipeline in an observational registry (ASPIRe) study, the complete occlusion rate was 79.0% (15/19) at 1 year follow-up. Eleven (5.8%) patients required retreatment [20]. Chalouhi et al. investigated in-stent stenosis in 139 patients treated using the PED (mean follow-up 6.7 months, range 3−24 months) and reported moderate/severe in-stent stenosis in 11 (7.9%) patients [21]. Martínez-Galdámez et al. recently reported an 81.8% total aneurysm occlusion rate at first-year follow-up in their prospective study titled the PFLEX Study with Shield Technology. The in-stent severe stenosis rate in that study was 3.1% [12]. In the IntrePED study, the neurological morbidity and mortality rate was 8.4%, the most common adverse event being ischemic stroke due to thromboembolic complications [2]. Two large metaanalyses of flow-diverter treatment demonstrated morbidity rates of 5.0%–7.3% and mortality rates of 2.8%–4.0% [17,18]. In the other study involving the PED Flex, the 30-day morbidity rate was 6.6%, with no deaths being reported [22]. One study with a large case series comparing the PED Flex with the new delivery system and the previous PED reported procedural success rates of 98% and 96%, respectively. The complication rate was significantly lower, and the rate of major morbidity or death was 5.6% for the previous PED cases and 1.9% for the PED Flex cases [11]. The delivery system of PED Flex provides an important innovation in light of the current studies with high procedural success rates and lower complication rates.

The limitations of this study include its retrospective design, small patient population, and relatively small mean aneurysm size.

The Pipeline embolization device has been renewed and improved. Our experience shows that the applicability and safety of the renewed delivery system offered by PED Flex for improvement of device apposition and opening are more beneficial than the first one in view of technical and clinical results. Although the new delivery system allows easier deployment, some technical aspects warrant a period of training as part of the learning process.
